# Dynamics of chromatin accessibility and gene regulation by MADS-domain transcription factors in flower development

**DOI:** 10.1186/gb-2014-15-3-r41

**Published:** 2014-03-03

**Authors:** Alice Pajoro, Pedro Madrigal, Jose M Muiño, José Tomás Matus, Jian Jin, Martin A Mecchia, Juan M Debernardi, Javier F Palatnik, Salma Balazadeh, Muhammad Arif, Diarmuid S Ó’Maoiléidigh, Frank Wellmer, Pawel Krajewski, José-Luis Riechmann, Gerco C Angenent, Kerstin Kaufmann

**Affiliations:** 1 Laboratory of Molecular Biology, Wageningen University, 6708PB Wageningen, The Netherlands; 2 Institute of Plant Genetics, Polish Academy of Sciences, 60-479 Poznań, Poland; 3 Max-Planck Institute for Molecular Genetics, Department of Computational Molecular Biology, Ihnestraße 63-73, 14195 Berlin, Germany; 4 Center for Research in Agricultural Genomics-CSIC-IRTA-UAB-UB, Campus UAB, 08193 Bellaterra, Barcelona, Spain; 5 IBR (Instituto de Biología Molecular y Celular de Rosario), Facultad de Ciencias Bioquímicas y Farmacéuticas, UNR, Suipacha 531, 2000 Rosario, Argentina; 6 Institute of Biochemistry and Biology, University of Potsdam, Potsdam 14476, Germany; 7 Trinity College Dublin, Smurfit Institute of Genetics, Dublin 2, Ireland; 8 Institució Catalana de Recerca i Estudis Avançats-ICREA, Barcelona 08010, Spain; 9 Business Unit Bioscience, Plant Research International, Wageningen 6700AP, The Netherlands

## Abstract

**Background:**

Development of eukaryotic organisms is controlled by transcription factors that trigger specific and global changes in gene expression programs. In plants, MADS-domain transcription factors act as master regulators of developmental switches and organ specification. However, the mechanisms by which these factors dynamically regulate the expression of their target genes at different developmental stages are still poorly understood.

**Results:**

We characterized the relationship of chromatin accessibility, gene expression, and DNA binding of two MADS-domain proteins at different stages of *Arabidopsis* flower development. Dynamic changes in APETALA1 and SEPALLATA3 DNA binding correlated with changes in gene expression, and many of the target genes could be associated with the developmental stage in which they are transcriptionally controlled. We also observe dynamic changes in chromatin accessibility during flower development. Remarkably, DNA binding of APETALA1 and SEPALLATA3 is largely independent of the accessibility status of their binding regions and it can precede increases in DNA accessibility. These results suggest that APETALA1 and SEPALLATA3 may modulate chromatin accessibility, thereby facilitating access of other transcriptional regulators to their target genes.

**Conclusions:**

Our findings indicate that different homeotic factors regulate partly overlapping, yet also distinctive sets of target genes in a partly stage-specific fashion. By combining the information from DNA-binding and gene expression data, we are able to propose models of stage-specific regulatory interactions, thereby addressing dynamics of regulatory networks throughout flower development. Furthermore, MADS-domain TFs may regulate gene expression by alternative strategies, one of which is modulation of chromatin accessibility.

## Background

Stem cells residing in meristems enable plants to produce new organs throughout their lives. Vegetative meristems in the shoot apex produce leaves, while reproductive meristems produce flowers or floral organs. The identities of different types of floral organs (sepals, petals, stamens, and carpels) are established by homeotic MADS-domain transcription factors (TFs) via modification of the leaf developmental programme [1]. Homeotic genes become activated in floral meristems through regulators that specify floral meristem identity. An important regulator of floral meristem identity in Arabidopsis is the MADS-box gene *APETALA1* (*AP1*), which has an additional role as homeotic regulator of sepal and petal identity [2]. Homeotic proteins specify different floral organ identities in a combinatorial fashion, mediated by protein interactions and formation of heteromeric quaternary protein complexes [3-5]. Homeotic genes can also enhance or repress each other’s expression, resulting in a complex transcriptional regulatory network. Mediators of higher-order complex formation are the largely redundantly acting members of the SEPALLATA MADS-domain subfamily, SEPALLATA 1 to 4 (SEP1-4) [1,6,7]. Therefore, these proteins have an important role in the specification of floral organ identities. Members of the MADS-domain TF family also act in many other developmental processes in plants, regulating directly and indirectly the expression of thousands of genes in the genome (for review, see [8,9]). Floral MADS-domain TFs are found in larger protein complexes together with chromatin remodeling and modifying proteins, as well as with general transcriptional co-regulators [5,10]. These interactions are important for the regulation of gene expression by the MADS-domain factors [5,10,11]. The expression of floral homeotic MADS-box genes is also regulated at the level of chromatin structure: outside the flower and at the earliest stages of floral meristem development, these genes are repressed by Polycomb group (PcG) protein complexes that act in concert with earlier acting MADS-domain TFs and other transcriptional regulators [12]. The physical and genetic interactions between MADS-domain proteins and chromatin regulatory factors suggest an important role of these TFs in controlling chromatin dynamics during plant development. To gain a genome-wide perspective on the developmental dynamics of gene regulation in plants, we studied MADS-domain TF occupancy, chromatin accessibility, and gene expression changes at different stages of Arabidopsis flower development. Our findings suggest that MADS-domain TFs may induce changes in chromatin accessibility, and thereby they are able to set appropriate chromatin landscapes for following regulatory processes leading to meristem and organ differentiation during flower development. By combining DNA-binding data and expression data, we established stage-specific gene regulatory interactions in floral morphogenesis.

## Results

### Developmental dynamics of floral gene regulation

**Figure 1:**
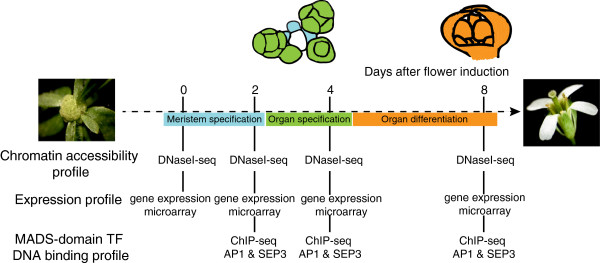
**Overview of the experimental set-up.** Using a system for synchronized floral induction (*pAP1*:AP1:GR *ap1 cal*), different developmental stages were analyzed: meristem specification (stage 2; 2 days after induction), organ specification (stage 4-5; 4 days after induction), and organ differentiation (stage 7-8; 8 days after induction). Around day 4, organ identity genes specify the floral whorls within the meristem, and sepal growth has been initiated. At day 8, sepals are largely differentiated, and the organs in the inner whorls are being formed. The experimental techniques used at each time point are indicated in the lower part of the figure. For illustrative purposes, images of wild-type floral meristems of the respective stages (color) are indicated above the graph.

We studied global changes in chromatin accessibility, gene expression, and DNA binding of two MADS-domain TFs at different stages of flower development (Figure 1). To obtain sufficient stage-specific plant material, we used an inducible system for synchronized flower development based on a chemically inducible version of the AP1 TF expressed under the control of its own promoter in the *ap1 cal* mutant background (*pAP1*:AP1-GR *ap1 cal* line). We analyzed different floral stages during which floral meristem specification (days 0 to 2), floral organ specification (day 4), and floral organ differentiation (day 8) take place [13]. In order to study chromatin accessibility at these different stages, we made use of DNase-seq [14]. Furthermore, we performed ChIP-seq experiments to identify stage-specific DNA-binding sites (BSs) of the two MADS-domain TFs, AP1 and SEP3. *SEP3* is a direct target gene of AP1 and becomes strongly expressed around floral stage 3, when the sepal primordia arise (day 3 after floral initiation) [15]. Genome-wide expression analyses were performed in order to detect changes in gene activity between different floral stages.

**Figure 2:**
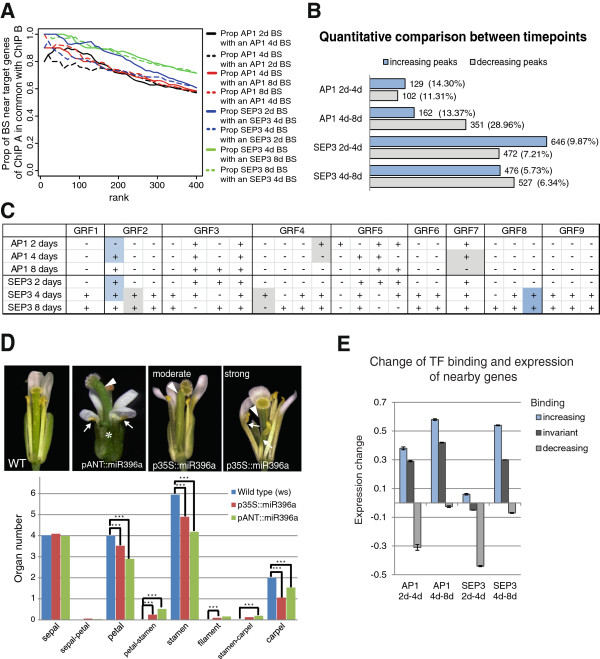
**Developmental dynamics of MADS-domain TF-bound genomic regions. (A)** Proportion of overlapping AP1 or SEP3 BSs between time points depending on their rank (1 = highest rank). Only peaks near genes (3 kb upstream to 1 kb downstream of the gene) were considered. **(B)** Changes in AP1 and SEP3 BSs between consecutive time points. ‘Increasing peaks’ and ‘decreasing peaks’ are genomic regions with a peak score at least two-fold higher or two-fold lower, respectively, when compared with the previous time point. Only significant peaks (FDR <0.001) near genes in at least one time point are considered. **(C)** Summary of AP1 and SEP3 BSs at GRF loci. Each locus has a number of columns depending on the number of different AP1 or SEP3 BSs at any time point. For each column, ‘–’ indicates that the region was not bound and ‘+’ that it was bound (FDR <0.001); two consecutive time points are colored in gray when the ChIP-seq score of the earlier time point is at least two-fold higher than at the later time point and in blue when it is two-fold lower. **(D)** Floral phenotypes of plants expressing miRNA396 from the 35S or p*ANT* promoter. One sepal and petal were removed for visualization. Arrow indicates petal-stamen organs, asterisk indicates conversion of floral organs into filament, arrowhead indicates ovary composed of a single valve in mutant flowers. In the column chart, data are represented as means, 100 flowers of each genotype were assessed. *** indicates significant difference at *P* value <0.001 by unpaired Student’s *t* test. **(E)** Mean change of log10 fold expression of genes in vicinity (up to 1 kb upstream of start or inside the gene) of different classes of AP1- and SEP3-bound genomic regions. Only differentially expressed genes were used (Additional file 7: Table S4). Bars correspond to standard error of mean.

The ChIP-seq experiments generated high confidence sets of TF-bound regions for each factor and time point (see Additional file 1: Table S1 for an overview and a list of TF-bound regions). Many TF binding events were common to the different time points (Figure 2A), this result also holds when we analyzed each biological ChIP-seq replicate independently (see Additional file 2: Figure S1). For example, 67% of AP1 target genes and 90% of SEP3 target genes identified at day 4 are also present in the day 8 dataset (see Additional file 3: Figure S2A). We also observed a number of stage-specific binding events and potential direct target genes, for example 21% of putative AP1 target genes at day 4 were not found at any other time point (see Additional file 3: Figure S2A). DNA binding of TFs is not a none-or-all phenomenon; rather, quantitative differences in TF occupancy can influence transcriptional behavior [16]. Therefore, we studied quantitative changes in AP1 and SEP3 binding levels between different time points [17,18]. By comparing ChIP-seq peak scores as a measure of relative binding levels, we identified several hundred genomic regions with changes in TF occupancy (fold-change (FC) ≥2; Figure 2B and see Additional file 4: Table S2). In case of significant AP1 binding sites, 26% show differences between days 2 and 4, and 42% between days 4 and 8. For SEP3 binding levels, 1,118 (17%) genomic regions showed changes between days 2 and 4 and 1,003 (12%) between days 4 and 8 (Figure 2B).

To investigate whether differences in AP1 and SEP3 binding are associated with stage-specific functions of these TFs, we analyzed the over-representation of GO categories in the different datasets. GO enrichment analysis revealed that genes involved in pattern formation, meristem maintenance, organ formation, and polarity are mostly bound by AP1 and SEP3 at early developmental stages (see Additional file 3: Figure S2B). For example, *STERILE APETALA* (*SAP*), a regulator of floral organ patterning [19], and *FILAMENTOUS FLOWER* (*FIL*) [20] and ASYMMETRIC LEAVES 1 and 2 [21], genes controlling axis specification, are among those genes. On the other hand, genes involved in hormonal signaling are more strongly bound at later developmental stages (see Additional file 3: Figure S2B). The results of stage-specific ChIP-seq experiments, in combination with gene expression data, therefore allow to identify stage-specific regulatory interactions.

Among the potential direct target genes of AP1 and SEP3, there is over-representation of specific TF families, and the degree of over-representation for a given family may vary between time points (see Additional file 5: Table S3), suggesting stage-specific regulatory interactions. A family that is over-represented among both AP1 and SEP3 targets at 2, 4, and 8 days (*P* value <0.05) is the GROWTH REGULATING FACTOR (GRF) family (see Additional file 5: Table S3). In particular, all nine GRF family genes are significantly bound by SEP3 (FDR <0.001), although a quantitative difference in binding levels was observed, and five of them are bound by AP1 (Figure 2C). *GRF* genes have well-known roles in leaf growth [22], but no known function in the determination of flower organ identity. Seven out of the nine Arabidopsis *GRF* genes (*GRF1*, *2*, *3*, *4*, *7*, *8*, and *9*) contain a target site for miR396 [22,23]. The floral phenotypes of plants overexpressing miR396a from the 35S or p*ANT* promoters largely resemble the phenotype of a weak *ap1* mutant allele, *ap1*-*3*, suggesting a role of these genes downstream of AP1. In *ap1*-*3* flowers, as well as in miR396a overexpression lines (Figure 2D), the second floral whorl is often occupied by petal-stamen mosaic structures [2,24]. Plants overexpressing miR396a show also a reduction in carpel number (Figure 2D). Severity of the mutant phenotype directly correlates with the level of reduction in GRF transcript abundance (Figure 2D and see Additional file 3: Figure S2C). In summary, these results indicate that, apparently redundant GRF family members are regulated in different ways, and that the phenotype that was observed in the miRNA-directed knockdown lines probably reflects the combined function of these family members in floral meristem patterning and in floral organ differentiation.

We next investigated the relationship at genome-wide level between changes of MADS-domain TF binding and changes in the expression of closely adjacent genes (that is, genes with a binding site within a region 1 kb upstream of the start of the gene or inside the gene) (Figure 2E). We observed a correlation between changes in binding and changes in expression. Genes located near regions with decreasing TF binding preferentially showed a reduction in their expression level, whereas increased TF binding was associated with an increase in the expression of nearby genes (Figure 2E).

In summary, AP1 and SEP3 binding sites overlap substantially between time points, but there is also an important number of BSs specific for each TF at each time point. Moreover, we observed that dynamic changes in AP1 and SEP3 DNA-binding correlate with changes in gene expression.

### Overlap and differences between AP1 and SEP3 DNA binding and potential direct target genes

**Figure 3:**
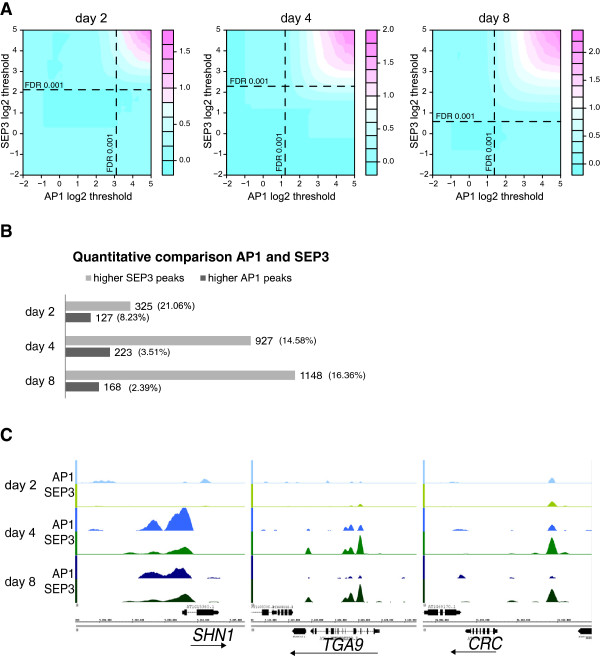
**Overlap and differences between AP1 and SEP3 DNA binding. (A)** Overview of AP1 and SEP3 common target genes obtained from the ChIP-seq datasets at the same time points. The figure shows the ratio between the observed number of common target genes divided by the expected number when the location of AP1 and SEP3 BSs are independent, this expected number was estimated by multiplying the proportion of AP1 BSs by the proportion of SEP3 BSs and by the total number of BSs. The x-axis and y-axis represent the threshold values for declaring a given region as significantly bound by AP1 and SEP3, respectively. **(B)** Changes in AP1 and SEP3 binding at common time points. ‘Higher AP1 peaks’ are genomic regions with AP1 peaks that are at least two-fold higher than the SEP3 peaks, while ‘higher SEP3 peaks’ are genomic regions with AP1 peaks at least two-fold lower than the SEP3 peaks. Only significant peaks (FDR <0.001) located in a region comprising 3 kb upstream and 1 kb downstream of a gene are considered. (Additional file 2: Table S2). **(C)** AP1 and SEP3 binding profiles for selected target genes. *SHINE 1* (*SHN1*) is an example of an AP1 target gene that is most strongly bound at day 4, whereas *TGA9*, a gene involved in anther development, and *CRC*, a gene involved in carpel development, are preferentially SEP3 targets.

We found a significant overlap for AP1 and SEP3 target genes (Figure 3A and see Additional file 6: Figure S3A), which is in agreement with previous observations that were made using different plant materials, antibodies for the AP1-GR fusion protein, and time points [15]. In agreement with the fact that SEP3 and AP1 form higher-order protein complexes with the B-class homeotic proteins APETALA3 (AP3) and PISTILLATA (PI), we observed a clear overlap between sets of potential direct target genes (see Additional file 6: Figure S3B) [25].

Results from Drosophila have shown that while many TFs have common binding sites in the genome, quantitative differences in binding levels correlate with the specific biological functions of different factors [26]. Quantitative comparison of genomic regions that are bound by both AP1 and SEP3 at the same time point shows that between 70% and 80% of the regions have peaks of similar height for both TFs (see Additional file 4: Table S2). Nevertheless, depending on the time point, from about 8% to 2% of all bound regions are preferentially bound by AP1 while a higher number of regions are more strongly occupied by SEP3 (FC ≥2; Figure 3B). For example, *SHN1*, a regulator of epidermal cell morphology of floral organs [27], is preferentially bound by AP1 at day 4. In contrast, *CRABS CLAW* (*CRC*), which is involved in specifying abaxial cell fate in carpels and in nectary formation [28], and *TGACG (TGA) MOTIF-BINDING PROTEIN 9* (*TGA9*), which is involved in anther formation [29] are preferentially bound by SEP3 (Figure 3C). These genes are significantly upregulated throughout all stages of flower development in the gene expression microarray data (see Additional file 7: Table S4). We confirmed the microarray results by quantitative PCR (qPCR) (see Additional file 6: Figure S3C). Thus, differences in quantitative levels of TF occupancy may help to explain target-gene specificity of floral homeotic protein complexes.

### Dynamics of chromatin accessibility during flower development

**Figure 4:**
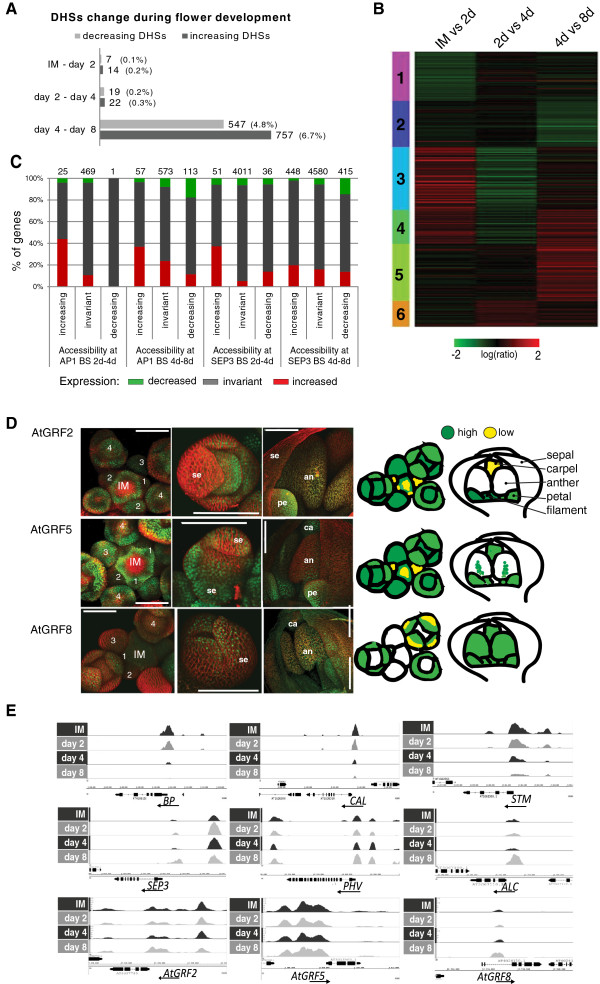
**Dynamics of chromatin accessibility in *Arabidopsis *flower development. (A)** Quantitative changes in DHSs between IM and day 2, 4, and 8 after flower induction for genomic regions detected as open chromatin at any time point and located nearby genes (3 kb upstream to 1 kb downstream of a gene). **(B)** K-means cluster analysis of differentially expressed genes. All the genes detected as differentially expressed (BH <0.05 and FC >1.8) in at least one time point comparison (IM *vs.* 2d, 2d *vs.* 4d, and 4d *vs.* 8d) are represented. **(C)** Percentage of genes in vicinity of AP1 or SEP3 and different classes of DHS, classified according to their expression change between days. Numbers above the bars show the total number of genes in the group. In all four cases there is a significant change of fractions across categories of DHS (χ2 test, *P* <0.001). **(D)** Confocal images of expression patterns of *pGRF*::*GRF-GFP* fusions in inflorescence meristems and during flower development. Expression patterns are summarized in schematic drawings on the right. Numbers indicate floral stage according to Smyth et al. [13], IM: inflorescence meristem, se: sepal, pe: petal, an: anther and ca: carpel. **(E)** Examples of TF gene loci that have DHSs with decreasing accessibility (top) and increasing accessibility (center) after flower induction. Genes involved in meristem identity like *STM*, *BREVIPEDICELLUS* (*BP*) and *CAL* show decreasing DHS peaks. On the other side, genes involved in flower organ initiation and determination like *SEP3*, *PHV*, and *ALC* show increased DHSs. In the bottom part of the figure are shown the accessibility profiles for *AtGRF2*, *AtGRF5*, and *AtGRF8* loci are shown. DNase I hypersensitivity profiles at *AtGRF2* and *AtGRF5* loci do not change during time while an increase in accessibility is found for *AtGRF8* locus between day 4 and 8.

Mapping of DNase I hypersensitive sites (DHSs) is a well-established method to identify the location of active gene regulatory elements [30]. The DNase I enzyme preferentially digests DNA in regions of low nucleosome occupancy, and DNase I digest of chromatin followed by deep sequencing identifies open or accessible genomic regions at genome-wide scale. DHSs have been found to be correlated with genomic regulatory features such as transcription start sites (TSSs), enhancers, and TF binding sites [31]. Focusing on genomic regions nearby genes (3 kb upstream of the start of the gene and 1 kb downstream of the end of the gene), we found that the overall number of detected high-confidence (FDR <0.01) DHSs at the different time points after AP1 induction vary between 5,680 and 8,789 (see Additional file 8: Table S5). We observed a high overlap (98.7%) between the DHSs identified at day 8 compared with the 41,193 previously identified DHSs in wild-type inflorescences (stages 1 to 11) [11], the larger number of DHSs in wild-type inflorescences may be a consequence of using tissue that represents a mixture of different stages. Whereas the majority of DHSs were invariant across consecutive time points (FC < √2), 1,370 quantitative changes in chromatin accessibility (measured as changes in DHS peak score) were detected. While there were only a small number of changes in DHS peak score comparing the different meristematic stages, the transition to organ differentiation (day 4 to 8) was found to be associated with the most changes in chromatin accessibility. There were significantly more differences between day 4 to 8 than between the earlier time points (*P* <2.2e-16, χ^2^ test) (see Additional file 9: Table S6). A total of 1,304 DHSs (11.8% of all DHSs detected at days 4 and 8) show quantitative differences in DHS peak score between days 4 and 8, with a slight preponderance of changes leading to increased accessibility (Figure 4A). Distinct clusters of differentially expressed genes were identified: those specific to early meristematic stages (clusters 1 and 2), transiently activated (cluster 6) or repressed (cluster 4) and genes that are specific to later floral stages (cluster 5) (Figure 4B). The trends in gene expression are reflected in concordant changes in chromatin accessibility: for example, genes that are expressed predominantly during meristematic stages of flower development (cluster 2), show over-representation of decreasing DHSs towards later stages (day 8). On the other hand, genes that are specifically activated later during floral organ development (cluster 5) show preferentially concordant increase in accessibility (see Additional file 10: Figure S4A). These data support the idea that changes in accessible genomic regions are linked with different sets of genes being active in meristematic cells *versus* differentiating tissues.

Next, we studied the relationship between changes in accessibility level of AP1- or SEP3-bound regions and expression of closely adjacent genes. Change in chromatin accessibility between meristematic tissues and differentiating floral organs is related with a corresponding change of expression of nearby genes (Figure 4C). This relation is statistically significant for both AP1- and SEP3-bound loci comparing days 2 to 4 and days 4 to 8 (*P* <0.001; χ2 test), where the proportions of upregulated genes are larger for regions with increased accessibility, and the proportions of downregulated genes are correspondingly smaller. Using members of the GRF family as an example, we analyzed how variations in chromatin accessibility were associated with differences in spatiotemporal gene activity. *GRF8* shows an increased SEP3 BS between days 4 and 8 and GRF8 chromatin becomes more accessible in differentiating floral organs (day 8) (see Additional file 9: Table S6). GFP reporter gene analyses show that the GRF8 protein is, in contrast to other factors such as GRF2 and 5, not expressed in flower meristems, and its expression increases in differentiating organs (Figure 4D,E).

General meristematic regulators are found among genes with a decrease in both accessibility and expression, such as *SHOOT MERISTEMLESS* (*STM*) (Figure 4E). These data are consistent with previous findings, which report *STM* expression mainly in meristems, while the expression is later restricted to cells in the gynoecium, which give rise to ovules [32]. A decrease in chromatin accessibility and expression is also found for loci that control early patterning processes in floral meristems, such as *AINTEGUMENTA-LIKE 6* (*AIL6*), *CAULIFLOWER* (*CAL*) (Figure 4E), and *STERILE APETALA* (*SAP*). These data are corroborated by previous studies that reported predominant expression of *AIL6*[33], *SAP*[19], and *CAL*[34] in meristems and young developing floral organ primordia (see Additional file 9: Table S6 and see Additional file 7: Table S4). Among the genes that show an increase in accessibility during flower development are a number of genes with specific roles in floral organ development, as well as more general regulators of organogenesis and growth. For example, the *SEPALLATA3* locus is among the earliest genes with increased accessibility (day 2). Other examples for genes with increased accessibility at day 4 include patterning genes like *PHAVOLUTA* (*PHV*) (Figure 4E). All these genes show a corresponding increase in expression. Among the genes that show predominantly increased accessibility from day 4 to day 8 (see Additional file 9: Table S6) are for example TFs known to be involved in the formation of carpels, ovules and seeds, like *ALCATRAZ* (*ALC*) and *NGATHA3* (*NGA3*) (Figure 4E and see Additional file 9: Table S6). In accordance with the idea that different promoter elements may control different aspects of gene regulation, we found that at a subset of those loci, individual DHSs change in opposite fashion: some DHS peaks increase, while others in the same promoter decrease (see Additional file 10: Figure S4B).

In summary, we found that changes in chromatin accessibility occur mainly between days 4 and 8 and that they correlate with changes in gene expression.

### Footprints of MADS-domain TF binding sites in flower development

**Figure 5:**
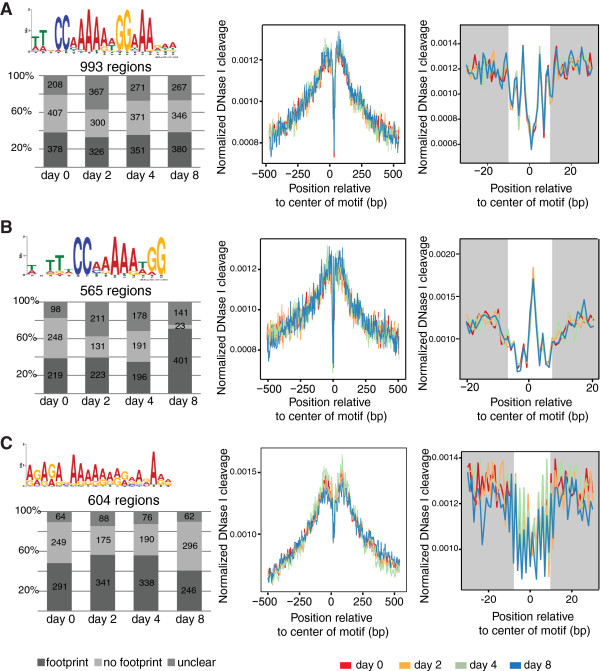
**DNase I footprints created by TF binding at different time points of flower development.** CArG box motifs were identified by MEME-ChIP in the AP1 and SEP3 peak regions (full list of motifs identified by MEME-ChIP in Additional file 3: Figure S4C). Footprints for selected motifs are shown in the right part of the Figure. **(A)** CArG-box 1 produces footprints at similar frequency at every time point. **(B)** CArG-box 2, identified only in the SEP3 ChIP-seq data, shows an increased footprint frequency at day 8. **(C)** An example of GA-rich motif, which produces more frequently footprints at early time points of flower development.

The binding of a TF protects the DNA from DNase I digestion, creating a specific ‘footprint’ [35]. We analyzed footprint patterns caused by protection of DNA upon AP1 or SEP3 binding. The time-series ChIP-seq data indicate that AP1 and SEP3 show quantitative differences in TF occupancy levels at different developmental stages (Figure 2). As MADS-domain TFs assemble into protein complexes in a combinatorial fashion, these differences may reflect changes in complex composition resulting in changes in DNA-binding specificity. In line with previous results [15,36], *de novo* identification of DNA sequence motifs in genomic regions bound by AP1 and SEP3 resulted mainly in motifs representing CArG boxes (see Additional file 10: Figure S4C). The generic CArG-box motif (hereafter named ‘CArG box 1’), which was identified both in the AP1 and the SEP3 datasets, possesses [A/T] stretches of variable length outside the central CC[A/T]_6_GG core. Thus, for AP1 and SEP3 we identify a longer consensus sequence than the canonical CArG-box motif: TTxCC[A/T]_6_GGxAA. A second CArG motif, lacking an [A/T] stretch on one side of the CArG-box, was identified in SEP3-bound regions (hereafter named ‘CArG box 2’). The generic CArG-box 1 has a footprint with a central dip corresponding to the region that is highly protected to the cutting of DNase I, indicating a possible contact between the protein and the nucleotide at that position (Figure 5A). In contrast, CArG-box 2 shows a footprint that suggests exposure of the DNA in the centre of the CArG-box (Figure 5B). By comparing the frequency of footprints at different developmental stages (Figure 5, left panels), we found that the genomic sequences corresponding to CArG-box 1 are similarly bound at all developmental stages. In contrast, those corresponding to CArG-box 2 show increasing frequencies of footprints at day 8 compared to earlier time points. This suggests that CArG-box 2 is more predominantly (though not exclusively) bound by SEP3 complexes lacking AP1 later in flower development. Indeed, among genes with the CArG box 2, we found an over-representation of GO categories involved in late reproductive processes, such as carpel, stamen, and anther development (see Additional file 10: Figure S4D). In summary, our data suggest that different CArG motifs are characterized by different footprint profiles and show temporal differences in their occupancy in flower development. The stage-specific enrichment of CArG motifs suggests a role in of combinatorial protein interactions in the spatiotemporal dynamics of AP1 and SEP3 DNA binding.

In agreement with previous findings [11,37], we also identified GA-rich sequence motifs in the genomic regions bound by AP1 and SEP3 (see Additional file 10: Figure S4C). Candidate proteins that bind to this motif are among others the BASIC PENTACYSTEINE (BPC) transcriptional regulators, which control multiple aspects of plant development [38]. Recently it was shown that BPC proteins interact with MADS-domain proteins to regulate their target genes [39]. For this motif, footprints are most frequently detected in the day 2 and 4 datasets (*P* ≤0.01, χ^2^ test), that is, during early stages of floral meristem development (Figure 5C). Thus, our data suggest a developmentally dynamic function of the GA-rich motif. However, its exact role and which factors bind to this motif remain to be determined.

### MADS-domain TF DNA binding precedes changes in chromatin accessibility

**Figure 6:**
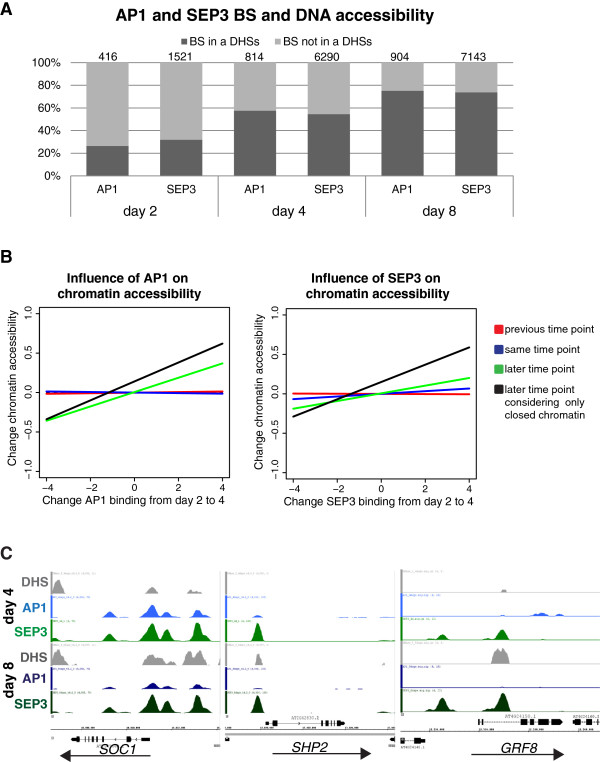
**MADS-domain TF binding determines chromatin accessibility changes. (A)** Overlap between AP1- and SEP3-bound genomic regions and DHSs at the different time-points after floral induction. Graph shows percentage of bound regions. Significant AP1 and SEP3 BSs located 3 kb upstream and 1 kb downstream of genes are considered. **(B)** Change in AP1 and SEP3 binding precedes change in chromatin accessibility. Regression lines with regression coefficients (Pearson correlation) between change in AP1 and SEP3 binding from day 2 to day 4 and change in DHSs between the different time points. A correlation is found only between change in AP1 and SEP3 binding from day 2 to day 4 and change in DHS from day 4 to day 8. The correlation is stronger when only closed regions (FDR >0.04) at day 4 are considered. Correlation is obtained considering AP1 or SEP3 BSs located in a range of 3 kb upstream and 1 kb downstream of at least one gene. **(C)** Examples of AP1 and SEP3 targets where DNA-binding events in closed chromatin at day 4 precede a more open chromatin state at the later stage.

In order to understand the dynamic relationship between chromatin accessibility and MADS-domain TF binding, we tested whether TF-bound genomic regions reside within DHSs (Figure 6). At the earliest time point after floral induction, day 2, the vast majority of AP1- and SEP3-bound regions (73% and 68%, respectively) do not reside in DHSs. However, the overlap increases at later time points as development progresses, associated with a generally higher number of detected DHSs at later time points (Additional file 8: Table S5). At day 4, over 50% of the sites bound by AP1 or SEP3 reside in DHSs, a fraction that increases to about 75% at day 8 (Figure 6A). We see a significant relation between change in binding and accessibility of sites between 4 and 8 days. This relation is mainly explained by an over-representation of sites with both decreased binding and decreased accessibility (see Additional file 11: Table S7).

Under the hypothesis that MADS-domain TFs have a role in the modulation of chromatin accessibility, we should expect that quantitative changes in MADS-domain TF DNA binding should precede corresponding changes in chromatin accessibility during development (but not *vice versa*). In agreement with this idea, we found that increase in levels of DNA binding by AP1 or SEP3 from day 2 to day 4 correlates more strongly with corresponding changes in chromatin accessibility from day 4 to day 8, rather than simultaneous changes in accessibility from day 2 to day 4 (Figure 6B and see Additional file 11: Table S7). The same result was observed when we analyzed each biological ChIP-seq replicate independently (see Additional file 12: Figure S5). This delay in change in chromatin accessibility suggests that MADS-domain TFs may act as pioneer factors [40] that directly or indirectly trigger changes in chromatin state during flower development. Among the genes for which AP1 and/or SEP3 may act as ‘pioneer factors’ are *SUPPRESSOR OF OVEREXPRESSION OF CO 1* (*SOC1*), *SHATTERPROOF 2* (*SHP2*), and *GRF8* (Figure 6C). In all three gene loci at day 4, regions are bound by AP1 and/or SEP3, while these regions are hardly or not accessible but become accessible at a later time point. *SOC1* is a special case since it is active in IMs, repressed in young floral meristems (stages 1 to 4) and later becomes expressed again in whorls 3 and 4, and it maintains expression during differentiation of stamens and carpels [41]. Also, the expression of *SHP2* and *GRF8* increases at later developmental stages (see Additional file 7: Table S4 and Figure 4D).

In conclusion, we observed that DNA-binding of AP1 and SEP3 can occur in chromatin regions that are not highly accessible, and that it can precede increase in DNA accessibility.

## Discussion

Plant development is controlled by the combined action of chromatin regulators and transcription factors. Here, we address the question of how this dynamic interplay is achieved at the molecular level using flower development as a model system. We characterize changes in MADS-domain TF occupancy, chromatin accessibility, and gene expression. Our results provide insights into the mechanisms by which MADS-domain TFs exert their master regulatory functions in meristem and organ differentiation in plants.

### Developmental regulation of gene expression at the chromatin level

Data from the animal field show that developmental control of gene expression is linked with dynamic changes in chromatin accessibility. Given that multicellular development originated independently in plants and animals, we aimed to understand how dynamic the chromatin accessibility landscape is during plant development, and how this reflects changes in developmental gene expression. In summary, we observed changes in chromatin accessibility in the course of flower development, mostly in the transition from meristematic stages to floral organ differentiation. These changes can reflect the establishment of multiple new cell types during flower differentiation, and be linked with the activation of regulatory regions driving cell-type specific expression patterns of genes. It can also be related to the fact that during floral organ morphogenesis, gene activation is more frequent than downregulation of genes [15,42]. Changes in DHSs globally correlate with changes in gene expression, although not all gene expression changes are associated with a change in chromatin accessibility. These findings suggest that there are multiple mechanisms by which developmental changes in gene expression are controlled, and that developmental changes in gene expression are partly manifested in changes in chromatin structure in plants.

### MADS-domain TFs regulate target gene expression in a dynamic fashion

Although many MADS-domain TF-bound regions are occupied by these factors throughout flower development, we did observe dynamic quantitative changes in occupancy levels at a number of binding sites. Binding site dynamics reflect regulatory dynamics of genes with stage-specific functions in flower development, such as floral meristem patterning and organ growth. In line with previous results [15,25,33], our data suggest that floral MADS-domain TFs can act as repressors or as activators of gene expression. Given that many genes show no quantitative change in MADS-domain TF binding but they are differentially expressed throughout flower development, it appears that MADS-domain TF binding alone *per se* is not sufficient to explain changes in their gene expression, or that there is a delay in the regulatory response, for example, due to the mechanisms by which gene expression is regulated. It is possible that promoter binding by MADS-domain TFs is a prerequisite for regulatory response, but that additional factors are needed to generate cell-type or stage-specific gene expression patterns. This finding is supported by the fact that SEP3 and AP1, like other MADS-domain TFs, show relatively broad expression patterns in meristems and developing floral organs, and are thereby expressed in a variety of cell types, while the gene expression patterns of their targets need to be more tightly controlled, as we could show for GRF genes.

### DNA-binding of MADS-domain TFs may trigger changes in chromatin accessibility

A result of the combined analysis of MADS-domain TF binding dynamics and chromatin accessibility is that MADS-domain TFs can select their binding sites independently of chromatin accessibility, and that binding of AP1 to DNA precedes local increase in chromatin accessibility. These results suggest that a mechanism by which AP1 regulates gene expression is through increasing accessibility of cis-regulatory regions. While this is the first report proposing such a mode of action for a plant TF, a similar mode of action has been previously described for animal TFs that trigger reprogramming of cell fate, such as Oct4, Sox2, Klf4, and c-Myc [43]. Previous results have shown that floral homeotic MADS-domain proteins form larger complexes together with ATP-dependent nucleosome remodelers and with histone-modifying enzymes *in planta*[5,11]. Taken together, MADS-domain proteins may act as ‘pioneer factors’ that trigger changes in chromatin accessibility. Given the important roles of MADS-domain proteins as master regulators of developmental switches and floral organ specification, this is an intriguing mode of action. But how do these proteins target different regulatory regions at different stages of development? Based on the different properties of CArG boxes that we found for SEP3 and AP1, we propose that different higher-order MADS-domain protein complexes have different affinities for specific ‘types’ of CArG boxes. Thereby, changing MADS-domain TF occupancy at individual sites could modulate chromatin accessibility in a stage- or organ-specific manner.

## Conclusion

**Figure 7:**
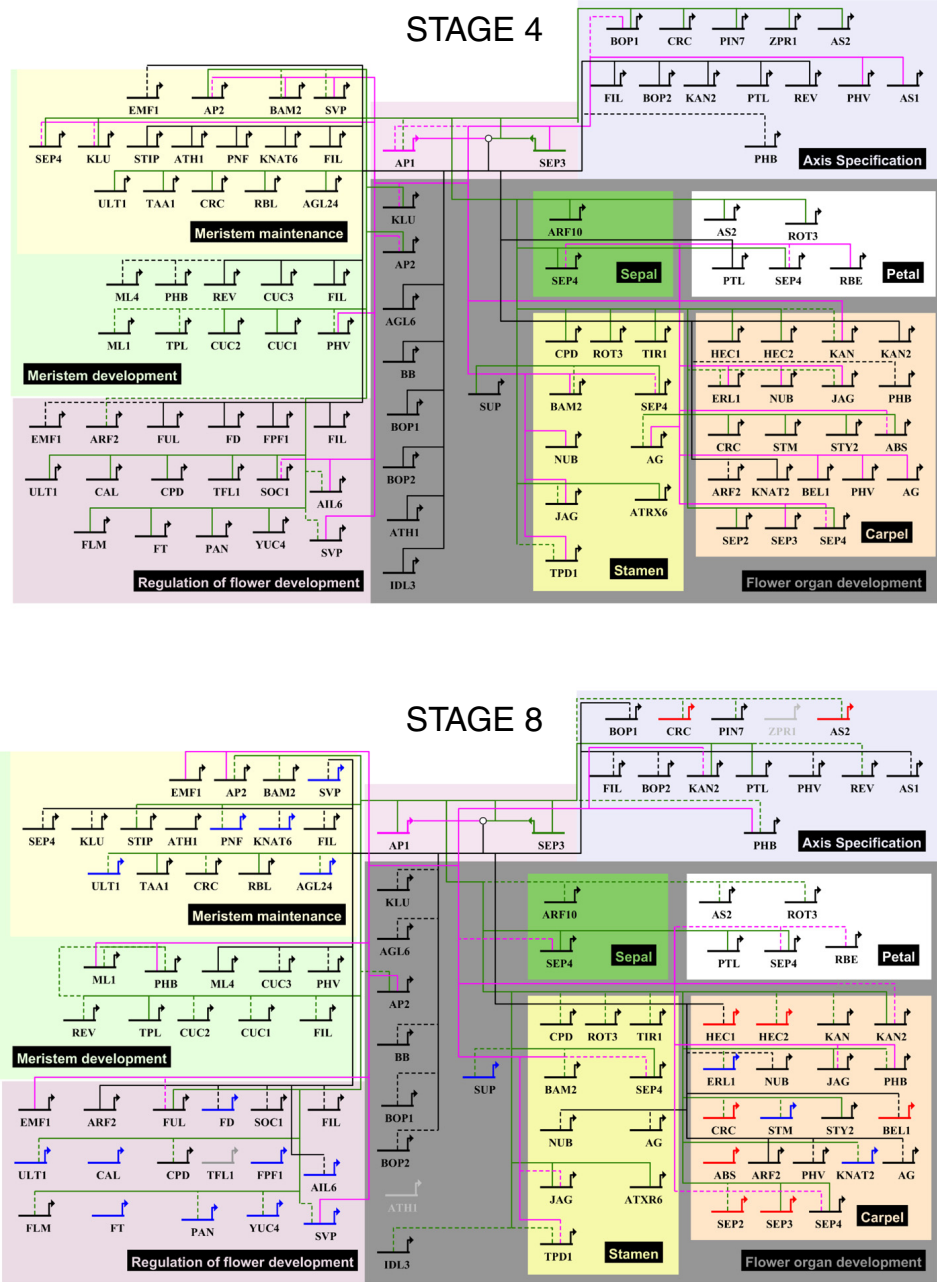
**Stage-specific regulatory networks.** Putative target gene networks at different floral stages reflecting preferential binding of AP1 and SEP3 at different time points (Figure 2) and making use of GO category enrichment analysis for differentially bound genes across the time points (Additional file 3: Figure S2B). Here, we focused on a selection of representative GO categories: meristem development, meristem maintenance, regulation of flower development, axis specification, and floral organ development (sepal, petal, stamen, and carpel development). We included only genes that belong to these categories and that were found to be preferentially bound by either AP1 or SEP3 on a comparison of floral stages 4 and 7/8 (corresponding to day 4 and day 8 in our data). Black line indicates common targets, while pink line indicates AP1-specific targets, and green line indicates SEP3 targets. Dashed lines are used to indicate gene with significant (FDR <0.001) TF-binding peak, while solid lines for genes with higher peak respectively at stage 4 or stage 8. In gray are genes not bound at the specific stage. In red are represented upregulated genes while in blue downregulated genes from day 4 to 8.

In conclusion, our work represents a first step to a better understanding of the dynamics of regulatory networks in plants. By combining the information from DNA-binding and gene expression data, we are able to propose models of stage-specific regulatory interactions (Figure 7). Our findings suggest that different homeotic factors regulate partly overlapping, yet also distinctive sets of target genes in a partly stage-specific fashion. Furthermore, MADS-domain TFs may regulate gene expression by alternative strategies, one of which is modulation of chromatin accessibility. Future research needs to reveal which target genes are specifically regulated by a certain homeotic protein complex, and by which exact molecular modes of action different sets of target genes can be modulated in specific ways.

## Materials and methods

### Plant material

All plants were grown at 20°C under long day condition (16 h light, 8 h dark). Plants for ChIP-seq and DNase-seq were grown on rock-wool, whereas plants for gene expression analysis were grown on soil.

### Tissue collection

For DNase-seq and ChIP-seq experiments: *pAP1*:AP1-GR *ap1*-*1 cal*-*1* plants were dipped after bolting (2 cm to 5 cm height) in the DEX- induction solution (2 μM Dexamethasone, 0.01% (v/v) ethanol, and 0.01% Silwet L-77) daily. First induction was performed 8 h after lights on and daily induction at 4 h after lights on. Material was collected before DEX-induction, as well as at 2 days, 4 days, and 8 days after the first treatment (8 h after lights on). Two biological samples were generated for each time point. For gene expression profiling experiments: approximately 4-week-old *pAP1*:AP1-GR *ap1-1 cal-1* plants were used. For each sample, inflorescence tissue from approximately 25 plants was collected using jeweler’s forceps as previously described [42]. Four biologically independent sets of samples were generated for each experiment. For induction, inflorescences were treated with a DEX-induction solution, or with an identical mock solution that lacked dexamethasone. Using plastic pipettes, the solutions were directly applied onto the inflorescences so that the cauliflower-like structures were completely drenched. As for the DNase-seq and ChIP-seq experiments, after the first induction, daily induction was performed 4 h after lights on, and material was collected at the corresponding time-point 8 h after lights on. Material was collected immediately after solution application (0 days, mock), and at 2 days, 4 days, and 8 days after the first treatment.

### DNase-seq experiments

Nuclei isolation was performed according to [44] with minor modifications. Tissue was ground in liquid nitrogen. For each time point, 0.2 g of plant material was used. Ground material was resuspended in 2 mL of cold modified Honda buffer (HBM: 25 mM Tris, 0.44 M sucrose, 10 mM MgCl2, 10 mM β-mercaptoethanol, 2 mM spermine, and 0.1% Triton) and filtrated through a 55 μm membrane. The membrane was washed with 1 mL HBM buffer. The filtrate was applied to a sucrose 2.5 M/40% Percoll gradient and centrifuged 30 min 2,500 × g at 4°C. Nuclei were collected in the interphase and washed with 10 mL cold HBB (HBM without spermine) and 10 mL cold HBC (HBB with 20% glycerol). Between each wash, nuclei were centrifuged for 10 min 1,000 × g at 4C. DNA digestion was performed according to [45] with minor modifications. Nuclei were resuspended in 2.5 mL buffer A (15 mM Tris-HCl (pH 8.0), 15 mM NaCl, 60 mM KCl, 1 mM EDTA (pH 8.0), 0.5 mM EGTA, 0.5 mM spermidine, and 11% sucrose) and divided into 12 1.5 mL tubes (aliquots of 200 μL). To each aliquot, 200 μL of 2× reaction buffer (Buffer A with 12 mM CaCl2, 150 mM NaCl) was added. Nuclei were mixed by inversion. DNase I was added (Roche Applied Science, Catalog #04716728001) to attain final concentrations of 110U-90U-70U-50U-35U-20U-15U-10U-7.5U-5U-2.5U-0U. Samples were incubated for 10 min at 37°C in a thermomixer. The DNase reaction was terminated by adding 400 μL of stop buffer (50 mM Tris-HCl (pH 8.0), 100 mM NaCl, 0.1% SDS, 100 mM EDTA (pH 8.0), 10 μg/mL Ribonuclease A, 1 mM spermidine, 0.3 mM spermine) and incubating at RT for 15 min. To each sample, 10 μL of 20 μg/mL proteinase K was added. After O/N incubation at 55°C, samples were centrifuged for 10 min at 13,000 × g. An aliquot of 10 μL of each sample were run on a 1% agarose gel. Samples that were not completely digested were selected for library preparation (Additional file 3: Figure S3E). DNA was precipitated by adding 0.9 volumes of isopropanol. The precipitated DNA was dried and left to resuspend in 100 μL HPLC water O/N at 4°C. DNA was purified with QIAGEN PCR purification kit (Cat. no. 28104). Two biological replicates for each time point were sequenced on Illumina HighSeq2000.

### ChIP-seq experiments

ChIP experiments were performed following a previously published protocol [46] using an anti-GR antibody (Glucocorticoid Receptor alpha Polyclonal antibody (PA1-516, Thermo Scientific), to precipitate AP1-GR), or a peptide SEP3 antibody [36]. 0.75 g of plant material were used for each biological replicate. ChIP experiments performed using pre-immuneserum were used as negative control for each time point. Two biological replicates for each experiment were sequenced on Illumina GAII or MiSeq.

### DNase-seq and ChIP-seq data analysis

Base calling was performed using CASAVA version 1.7 for AP1 4 and 8 days ChIP-seq experiments days, while CASAVA version 1.8 was used for all the other analysis. Sequence reads reported by the Illumina’s CASAVA v1.8 pipeline as low quality reads were removed from further analysis. CASAVA v1.7 does this automatically. FASTQ files were mapped to the Arabidopsis thaliana genome [47] using Bowtie [48] version 0.12.7, allowing up to three mismatches. Sequence reads mapped to mitochondrial and chloroplast chromosomes or mapping on multiple locations were removed. An overview of sequencing data is reported in Additional file 13: Table S8. Reproducibility between biological replicates was assessed using the Pearson correlation coefficient (PCC) for the genome-wide reads distribution at each pair of replicates on a single nucleotide resolution, for this, we used the script ‘correlation.awk’ provided by [17], the results were: PCC >0.99 for DNase-seq experiments, and 0.80 < PCC < 0.977 for ChIP-seq experiments. Because of the high reproducibility of the data, FASTAQ files for replicates of the same experiment were combined. We used MACS 2.0.10 [49] with default parameters except --mfold which was set to ‘2.20’) to identify significant BSs for ChIP-seq experiments and significant DNase I hypersensitive sites (DHSs) for DNase-seq experiments. We used a cutoff of FDR ≤0.01 and FDR ≤0.001 (--qvalue parameter in MACS) for DNase-seq and ChIP-seq experiments, respectively. Genomic regions were associated with genes if located 3 kb upstream of the start of the gene up to 1 kb downstream of the end of the gene using the function distance2Genes in the Bioconductor package CSAR [50] for genes annotated in TAIR10.

#### ***Quantitative comparison of ChIP-seq and DNase-seq experiments***

We followed the Bardet et al. [17] protocol for the peak alignment and normalization. Namely, we created an aggregated list of ChIP-seq and DHSs peaks in a region ± 75 bp around the peak summit, and then scored each one of those regions by the highest mapped read count normalized by total number of mapped reads in the library. This score was subsequently scaled by the score in the corresponding control sample in the same region. Quantile normalization implemented in the preprocessCore R package [51] was then applied independently to all DNase-seq and to all ChIP-seq score values.

Changes in DHSs and putative TF BSs across the different time stages were quantified by means of (fold-change ratio). We classified regions as invariant when the fold-change was ≤ √2 for DNase-seq data and ≤2 for ChIP-seq data. Otherwise the region was classified as being an increasing or decreasing region according to the sign of the log2.

The simultaneous analysis of dependence between chromatin accessibility changes and TF binding changes, and of the influence of these factors on changes in gene expression (Figures 2E, 4C) was done by the chi-square test in Genstat 15 [52]. DNA sequences and overlapping regions were extracted using BEDTools [53].

#### ***Motif analysis and DNase I cleavage***

For motif identification, sequences of ChIP-seq peaks ± 50 bp around the peak summits, were submitted to MEME-ChIP [54] after processed with RepeatMasker [55]; we used default parameters for MEME-ChIP except the motif site distribution (‘-mod’) parameter that was set to *any number of repetitions* (*anr*). Motif occurrences were found in TF BSs (located 3 kb up to 1 kb downstream of genes annotated in TAIR10) using FIMO [56] at *P* value <1e-5, and the DNase I cuts ± 100 bp around the motif matches at the same time stage were submitted to CENTIPEDE [57] together with the proximity to the nearest TSS and the FIMO log-likelihood score ratio to infer TF binding by digital genomic footprinting. Then, each site was classified according to its posterior probability (pp) into three classes: footprint (pp ≥0.9), no footprint (pp ≤0.1), and unclear bound state (0.1 < pp < 0.9). For visualization of the average DNase I cleavage in Figure 5 in a window ± 500 bp around the footprint, running-median smoothing was applied (width of median window equal to 5).

Information from The Plant Transcription Factor Database ([58]) was used to identify overrepresented TF families. GO over-representation analysis was performed using the Cytoscape plugin BINGO [59].

### RNA preparation for microarray experiments

Total RNA was isolated from tissue samples using the Plant Total RNA kit (Sigma-Aldrich) according to the manufacturer’s instructions. Quality of RNA samples was evaluated using a Bioanalyzer and a RNA Nano 6000 kit (Agilent). RNA concentrations were determined using a Nanodrop ND-1000 spectrophotometer (Thermo Fisher Scientific).

### Microarray set-up and experiments

Agilent microarrays were designed using the *e*Array software pipeline [60] and TAIR genome annotation v8, and contain probes corresponding to 28,327 annotated genes (see [15]. Microarrays were used following manufacturer’s instructions. RNA samples were labeled with fluorescent dyes using the Quick Amp Labeling Kit (Agilent). Microarray hybridizations (65 C, 16 h) and washes were performed with Agilent reagents and following standard protocols. Microarrays were scanned using an Agilent DNA Microarray Scanner, and data were acquired using Agilent’s Feature Extraction Software. Four independent sets of biological samples were used for the experiments. The dyes used for labeling RNA from a given time point were switched in the replicate experiments to reduce dye-related artifacts. Samples were co-hybridized as follows: 0 days to 2 days, 2 days to 4 days, and 4 days to 8 days, resulting in a total of three hybridizations per set, and two biological replicate sets labeled with each dye polarity.

### Gene expression microarray data analysis

Feature extraction software pre-processed data from the Agilent microarrays were imported into the Resolver gene expression data analysis system version 7.1 (Rosetta Biosoftware, Seattle, WA) and processed as described [42]. Resolver uses a platform-specific error model-based approach to stabilize the variance estimation to improve the specificity and sensitivity in differential gene expression detection [61]. The data from the four biological replicates of each condition were combined, resulting in an error-model weighted average of the four. The *P* values for differential expression calculated by Resolver were adjusted for multi-hypothesis testing using the Benjamini & Hochberg procedure, as implemented in the Bioconductor *multtest* package in R [62]. Genes for which the Benjamini & Hochberg-adjusted *P* value was <0.05 in at least one of the comparisons (that is, time points), and that passed an absolute fold-change (FC) cutoff of 1.8, were considered as differentially expressed (see Additional file 6: Table S4). Genes that were detected as differentially expressed were subjected to cluster analysis using the k-means algorithm implemented in Resolver (partitioning into different numbers of clusters was tested, and k = 6 was selected for producing the most consistent clusters (Figure 4B).

### Isolation of RNA and real-time PCR analysis

Total RNA was extracted using Invitek Kit according to the manufacturer’s protocol. DNase I digestion was performed on total RNA using DNase I from Invitrogen. RNA integrity was checked on 1% (w/v) agarose gels before and after DNase I treatment. Absence of genomic DNA was confirmed subsequently by qRT-PCR using primers, which amplify an intron sequence of the gene At5g65080 (Forward 5′-TTTTTTGCCCCCTTCGAATC-3′ and reverse 5′-ATCTTCCGCCACCACATTGTAC-3′). First-strand cDNA was synthesized from 4 μg of total RNA using TaqMan kit (Roche) cDNA Synthesis Kit following the manufacturer’s protocol. The efficiency of cDNA was estimated by qRT-PCR using two different primer sets annealing 5′- and 3′- ends of a control gene, glyceraldehyde-3-phosphate dehydrogenase (GAPDH) (At3g26650), respectively, (GAPDH3′: forward 5′-TTG GTG ACA ACA GGT CAA GCA - 3′ and reverse 5′-AAA CTT GTC GCT CAA TGC AAT C-3′) (GAPDH5′: forward 5′-TCT CGA TCT CAA TTT CGC AAA A - 3′ and reverse 5′-CGA AAC CGT TGA TTC CGA TTC -3′). Transcript levels of each gene were normalized to *ACTIN2* gene (5′- TCCCTCAGCACATTCCAGCAGAT-3′ and reverse 5′-AACGATTCCTGGACCTGCCTCATC-3′). Large-scale qRT-PCR for 1,880 TFs was performed as described previously [63,64], using an ABI PRISM 7900HT sequence detection system (Applied Biosystems Applera, Darmstadt, Germany). Amplification products were visualized using SYBR Green (Applied Biosystems).

### MIR396 constructs and GFP fusion reporter gene constructs

35S:miR396a was generated by fusing 400 bp of MIR396a precursor to the 35S promoter in the pCHF3 binary plasmid [65]. ANT: miR396a was generated by replacing the 35S viral promoter in the previous vector with the ANT promoter (5.8 kb upstream regulatory sequences) [66].

*AtGRF2*, *AtGRF5*, *AtGRF7*, and *AtGRF8* genomic regions were amplified by PCR using the following primers: AtGRF2, fw: 5′-AACATTTGGTTGGTAATGTCAGCGT-3′ rev: 5′-GGTTGTGTAATGAAAGTAATCGCCA-3′, AtGRF5, fw: 5′-GTATGTTCAAATAATGTGAATCGTGG-3′ rev: 5′-GCTACCTGAGAAAATAAATTTAAACT-3′ AtGRF7, fw: 5′-GAATCTTGTTCTTCAGAAAGATGAAC-3′ rev: 5′-AACCTGGCTGCTTTCGTCGGAC-3′ and, AtGRF8, fw: 5′-GTTTGTTTGTTACATTGCCGTTT-3′ rev: 5′-GCTTGAGCTTCTGCTGCA-3′. The PCR fragments were cloned into the GATEWAY vector pCR8/GW/TOPO from Invitrogen and transferred via LR reaction into the destination vector pMDC107 [67]. Expression vectors were introduced into *Arabidopsis thaliana* ecotype Col-0 by floral dip transformation [68]. Transformant plants were select on MS medium with Hygromycin (10 ug/mL).

### Confocal Scanning Laser Microscopy (CSLM)

GFP tagged protein localization was observed trough CSLM on Leica SPE DM5500 upright microscope using a ACS APO 40x/1.15 oil lens and using the LAS AF 1.8.2 software. FM4-64 dye was added to 0.1% agar at a concentration of 5 M and used as staining for cell membranes. GFP and FM4-64 dye were excited with the 488-nm line of an Argon ion laser. The GFP emission was detected at a bandwidth of 505-530 nm, while FM4-64 dye and chloroplast auto fluorescence were detected at a bandwidth of 650 nm. After acquisition optical slices were median filtered and three-dimensional projections were generated with LAS AF 1.8.2 software package.

### Accession numbers

Microarray data have been deposited with the NCBI Gene Expression Omnibus (GEO) under accession number GSE47981. ChIP-seq and DNase-seq data have been deposited under accession number GSE46986 and GSE46894, respectively.

## Abbreviations

AP1: APETALA1; BS: Binding site; ChIP-seq: Chromatin immunoprecipitation of DNA followed by DNA sequencing; DHS: DNase I hypersensitive sites; GRF: GROWTH REGULATING FACTOR; qPCR: quantitative PCR; SEP3: SEPALLATA3; TF: Transcription factor.

## Competing interests

The authors declare that they have no competing interests.

## Authors’ contributions

AP performed the ChIP experiments, DNaseI-seq experiments, generated and analyzed the marker lines, and drafted the manuscript. PM and JMM were responsible for bioinformatic analysis; AP and PM interpreted bioinformatics results; JTM and JJ performed the microarray studies and the data analysis; SB and MA helped with the transcriptome analysis; MAM, JMD, JP, and DSOM performed biological analysis; AP, FW, PK, and JLR participated in the design of the study and in the data analysis, and drafted the manuscript. GCA and KK conceived of the study, supervised and coordinated the study and drafted the manuscript. All authors carefully read and approved the final manuscript.

## Supplementary Material

Additional file 1: Table S1 ChIP-seq peak calling for AP1 and SEP3 ChIP-seq at different time points. The table shows peaks with FDR <0.001 and nearby genes for each dataset. Nearby genes are genes with the compared peaks 3 kb upstream of the start of the gene and 1 kb downstream of the end of the gene. In the overview table (sheet: overview) are summarized the total number of peaks for each dataset and the number of peaks nearby a gene for FDR <0.001. Click here for file

Additional file 2: Figure S1 Proportion of overlapping AP1 or SEP3 BSs between different time points depending on their rank (1 = highest rank) for pooled dataset and separate biological replicates. The figures were obtained in the same way like Figure 2A. We have performed the analysis for the same data as reported in the main manuscript **(A)** and for each replicate independently **(B, C)**, only analyzing replicates 1 for each experiment **(B)** or only analyzing replicates 2 for each experiment **(C)**. These figures shows that the rank-dependent pattern of overlap that we found is the same when combining the replicates or treating them independently. Click here for file

Additional file 3: Figure S2 MADS-domain TF binding dynamics. **(A)** Overview of AP1 and SEP3 ChIP-seq datasets from different time points. The number of target genes that were unique to, or shared across, the different time points is indicated (Additional file 1: Table S1). **(B)** Gene ontology enrichment for increasing and decreasing AP1- and SEP3-bound genomic regions. The heat map includes all overrepresented categories with at least five genes and *P* value <0.0001. Parental categories with more than 90% overlap with the child category have been removed. **(C)** AtGRF expression levels in plants overexpressing miR396a. Click here for file

Additional file 4: Table S2 ChIP-seq quantitative comparison between AP1 and SEP3 binding at different time points and between the two transcription factors at the same time point. The table shows the list of genomic regions that are increasing, decreasing, or invariant between the two compared time points. Only regions with a significant peak (FDR <0.001) in at least one of the two datasets compared are considered. Click here for file

Additional file 5: Table S3 Over-representation of TF families among significant (FDR <0.001) potential direct target genes of AP1 or SEP3 at different time points. TF families that are over represented (*P* value <0.05) among either AP1 or SEP3 targets in at least one time point are shown in the table. Click here for file

Additional file 6: Figure S3 AP1 and SEP3 specific binding. **(A)** Overview of AP1 and SEP3 ChIP-seq datasets from different time points. The number of BSs that were unique to, or shared across, the two TFs is indicated. **(B)** Venn diagrams show overlap in potential direct target genes (genes with peak between 3 kb upstream of the start of the gene and 1 kb downstream of the end of the gene) between AP1, SEP3, AP3, and PI ChIP-seq datasets. **(C)** qPCR results showing expression level at different time points for selected target genes in Figure 3. Click here for file

Additional file 7: Table S4 Genes identified as differentially expressed after AP1 activation. Microarray results show genes that are differentially expressed between IM and day 2, days 2 and 4, and days 4 and 8. Click here for file

Additional file 8: Table S5 DNase-seq peak calling for the different time points. The table shows DHSs with FDR <0.01 and nearby genes for each dataset. Nearby genes are genes with the compared peaks 3 kb upstream of the start of the gene and 1 kb downstream of the end of the gene. The total number of DHSs for each time point and the number of DHSs nearby a gene for FDR <0.01 are summarized in the overview table. Click here for file

Additional file 9: Table S6 DHS quantitative comparison between different time points. The table shows the list of genomic regions that are increasing, decreasing, or invariant between the two compared time points. Only regions with a significant DHS (FDR <0.01) in at least one of the two datasets compared are considered. Click here for file

Additional file 10: Figure S4 Chromatin accessibility and TF expression at different stages of flower development. **(A)** Venn diagram showing the distribution in the expression of cluster 2 and cluster 5 genes with increasing and decreasing DHSs between day 4 and day 8. **(B)** Venn diagram shows genes with increasing and decreasing DHSs between day 2 *vs.* 4 and day 4 *vs.* 8. Forty-six genes have both increasing and decreasing DHSs from day 4 to day 8. **(C)** Full list of motifs identified by MEME-ChIP in the AP1 and SEP3 peaks regions. Table shows consensus sequences and motifs based on position-specific probability matrices that were identified by MEME-ChIP, and TFs that potentially recognize those motifs identified by TOMTOM. **(D)** Gene ontology enrichment for SEP3-bound genomic regions at day 8 with CArG-box motif 1 and CArG-box motif 2. The graph shows terminal over-represented categories that belong to ‘biological regulation’ and ‘developmental process’. Only categories with at least five genes and *P* value <0.05 were considered. **€** Gel showing partially DNase I-digested chromatin that was submitted for sequencing. Click here for file

Additional file 11: Table S7 Number of genes in vicinity of different classes of BSs classified according to accessibility change. Numbers in yellow indicate cells in which significant deviations from independence are located. Click here for file

Additional file 12: Figure S5 Change in MADS-DNA binding precedes change in chromatin accessibility. The figures were obtained in the same way as for Figure 6B. The analysis was repeated for each replicate independently and for the combined analysis for both AP1 **(A)** and SEP3 **(B)**. The results and conclusions are similar in all cases. Click here for file

Additional file 13: Table S8 Overview of sequencing data. Total number of reads obtained with Illumina sequencing, aligned reads, and uniquely aligned reads for each sample. Click here for file
