# Supplementary figures and images for: Dynamics of chromatin accessibility and gene regulation by MADS-domain transcription factors in flower development
Source: Genome Biol. 2014 Mar 3;15(3):R41. doi: 10.1186/gb-2014-15-3-r41 (PMC4054849; doi:10.1186/gb-2014-15-3-r41)

**A**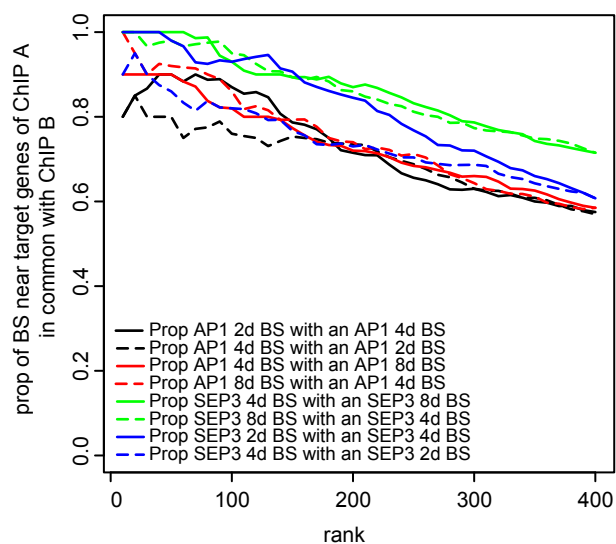**B**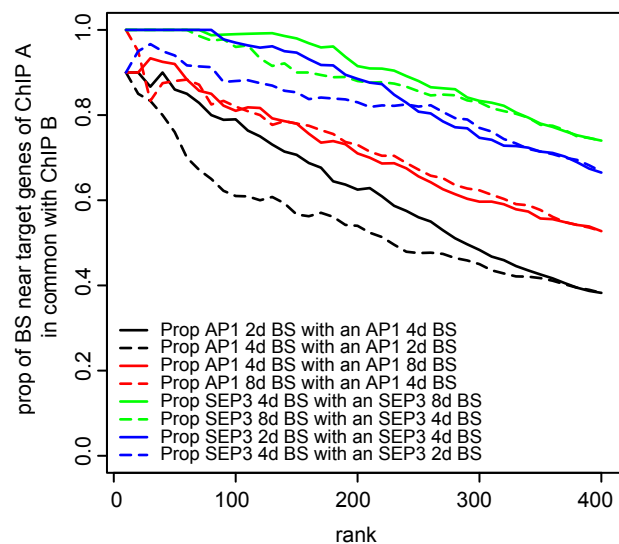**C**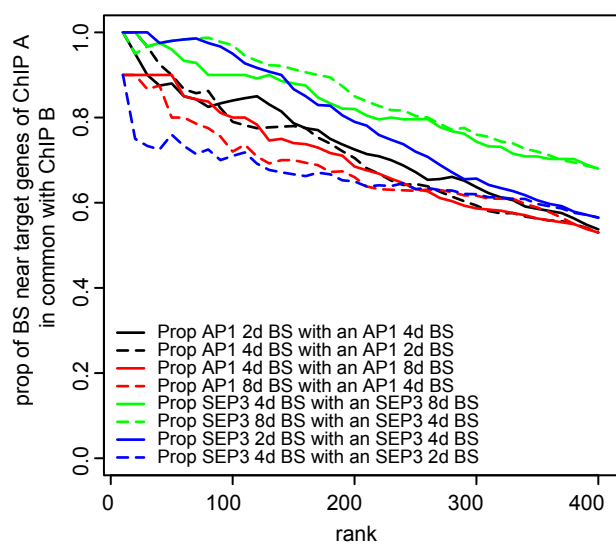

Supplement: Additional file 2: Figure S1 — Proportion of overlapping AP1 or SEP3 BSs between different time points depending on their rank (1 = highest rank) for pooled dataset and separate biological replicates. The figures were obtained in the same way like Figure 2A. We have performed the analysis for the same data as reported in the main manuscript (A) and for each replicate independently (B, C), only analyzing replicates 1 for each experiment (B) or only analyzing replicates 2 for each experiment (C). These figures shows that the rank-dependent pattern of overlap that we found is the same when combining the replicates or treating them independently. [file gb-2014-15-3-r41-S2.pdf]

**A**

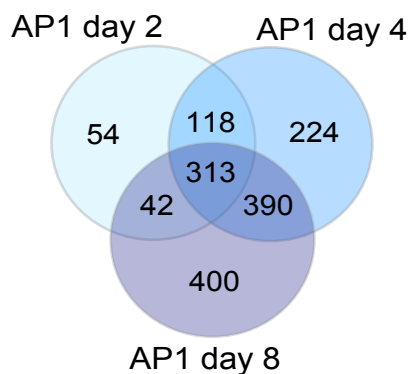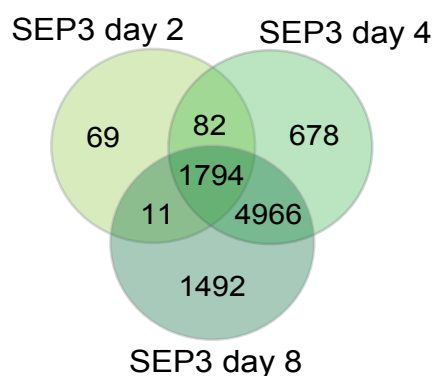

**B**

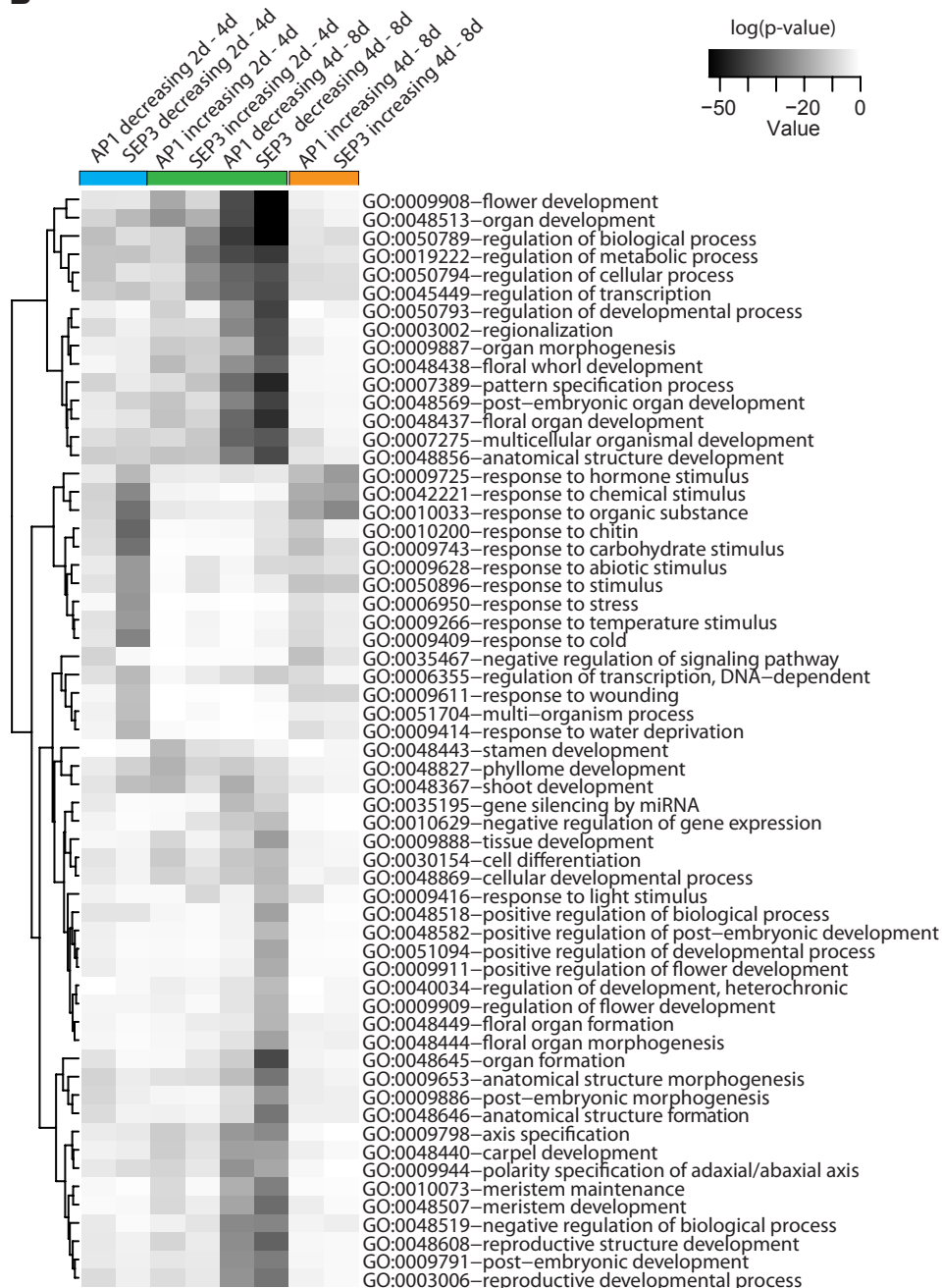

**C**

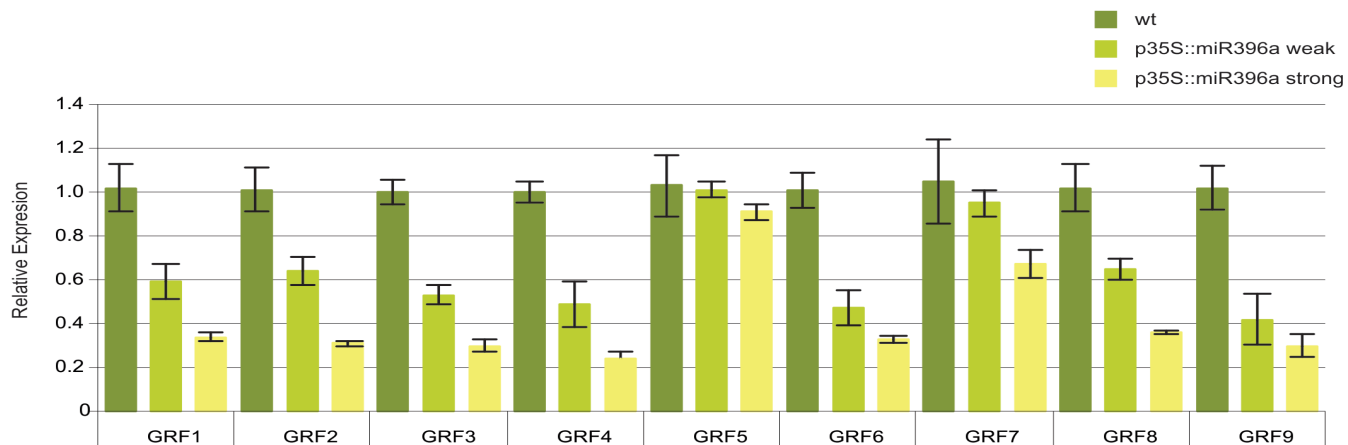

Supplement: Additional file 3: Figure S2 — MADS-domain TF binding dynamics. (A) Overview of AP1 and SEP3 ChIP-seq datasets from different time points. The number of target genes that were unique to, or shared across, the different time points is indicated (Additional file 1: Table S1). (B) Gene ontology enrichment for increasing and decreasing AP1- and SEP3-bound genomic regions. The heat map includes all overrepresented categories with at least five genes and P value <0.0001. Parental categories with more than 90% overlap with the child category have been removed. (C) AtGRF expression levels in plants overexpressing miR396a. [file gb-2014-15-3-r41-S3.pdf]

**A**

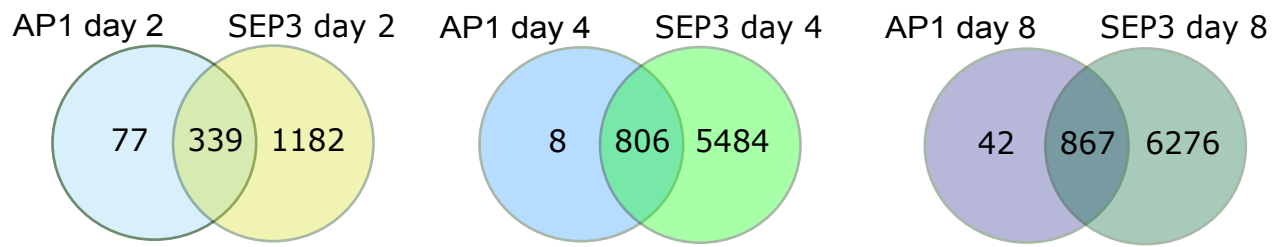

**B**

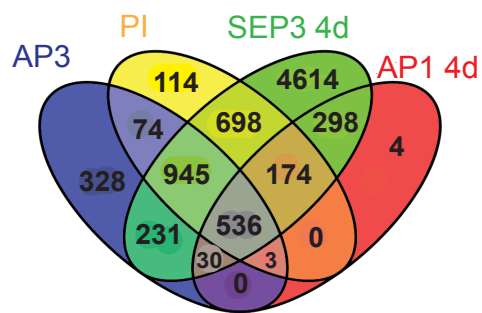

**C**

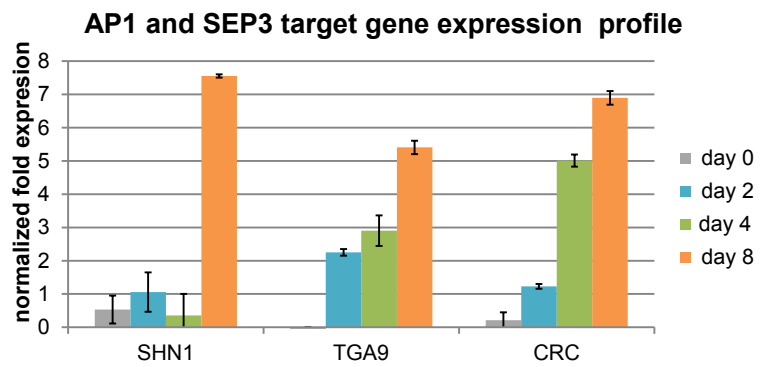

Supplement: Additional file 6: Figure S3 — AP1 and SEP3 specific binding. (A) Overview of AP1 and SEP3 ChIP-seq datasets from different time points. The number of BSs that were unique to, or shared across, the two TFs is indicated. (B) Venn diagrams show overlap in potential direct target genes (genes with peak between 3 kb upstream of the start of the gene and 1 kb downstream of the end of the gene) between AP1, SEP3, AP3, and PI ChIP-seq datasets. (C) qPCR results showing expression level at different time points for selected target genes in Figure 3. [file gb-2014-15-3-r41-S6.pdf]
