# Supplementary material for: Dynamics of chromatin accessibility and gene regulation by MADS-domain transcription factors in flower development
Source: Genome Biol. 2014 Mar 3;15(3):R41. doi: 10.1186/gb-2014-15-3-r41 (PMC4054849; doi:10.1186/gb-2014-15-3-r41)

A

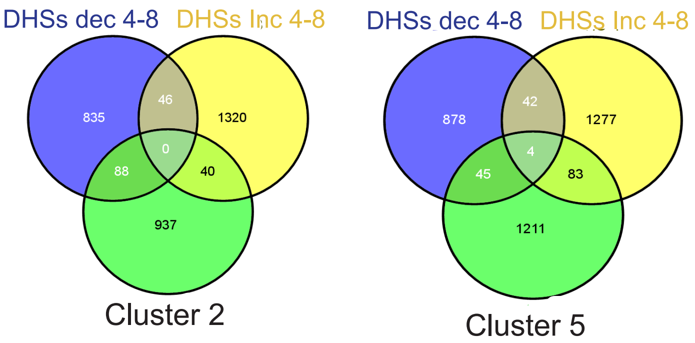

B

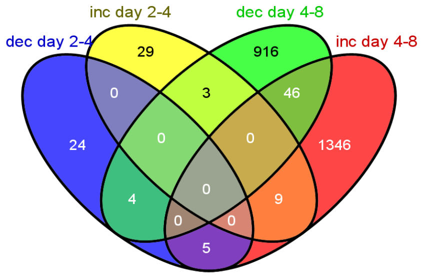

C

Consensus	Motif	Identified TFs (TOMTOM, only plants)
**AP1 day 2**		
GAGAAAGAGAGAGAG	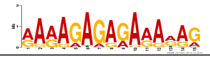	MA0120.1 (id1)
TTACCAAAAAAGGAA	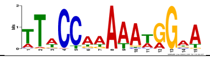	MA0082.1 (SQUAMOSA)<br>MA0001.1 (AGL3)<br>MA0005.1 (AG)
TTTTTTTGTTTGTCTCTTT	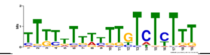	MA0120.1 (id1)<br>MA0045.1 (HMG-I/Y)<br>MA0082.1 (SQUAMOSA)
TGTCACGTCCCATC	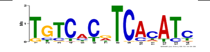	MA0129.1 (TGA1A)<br>MA0097.1 (bZIP911)<br>MA0096.1 (bZIP910)
**AP1 day 4**		
CCAAAAAAGGAAAA	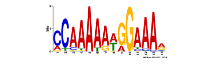	MA0082.1 (SQUAMOSA)<br>MA0045.1 (HMG-I/Y)<br>MA0001.1 (AGL3)<br>MA0005.1 (AG)
TTTCTTCTTTTTTTTCTCTCT	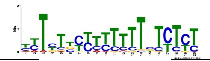	MA0045.1 (HMG-I/Y)
AAAGAGAGAAAAAAA	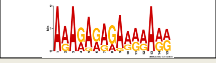	-
**AP1 day 8**		
TTTTTTTTTTCCTTTTTTGG	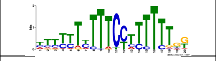	MA0082.1 (SQUAMOSA)
TTCCATTTTTGGAAA	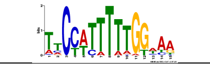	MA0082.1 (SQUAMOSA)<br>MA0001.1 (AGL3)<br>MA0005.1 (AG)
ATTGGACCACA	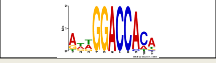	-
**SEP3 day 2**		
CCAAAAAAGGAA	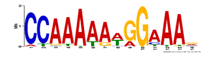	MA0082.1 (SQUAMOSA)<br>MA0001.1 (AGL3)<br>MA0045.1 (HMG-I/Y)<br>MA0005.1 (AG)
GGAAAAAAAAGAAGAGAAAG	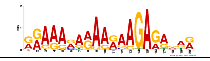	MA0120.1 (id1)
TTTCCTAAAATGG	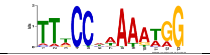	MA0001.1 (AGL3)<br>MA0082.1 (SQUAMOSA)<br>MA0005.1 (AG)
TTTTCTTGTTTTTGTTTCTT	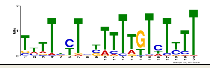	MA0045.1 (HMG-I/Y)<br>MA0082.1 (SQUAMOSA)<br>MA0005.1 (AG)
**SEP3 day 4**		
TTACCAAAAAAGGAAAGAAA	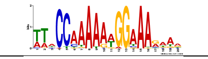	MA0082.1 (SQUAMOSA)<br>MA0005.1 (AG)
CTTCTTTTTCTTTCTTTTTT	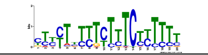	-
TTTCCAAAAATGG	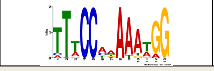	MA0001.1 (AGL3)<br>MA0005.1 (AG)<br>MA0082.1 (SQUAMOSA)
**SEP3 day 8**		
TTACCAAAAAAGGAAAGAA	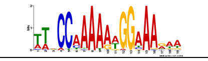	MA0082.1 (SQUAMOSA)<br>MA0005.1 (AG)
AAAAAAGAAAGAGAGAGAAA	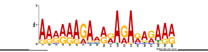	-
TATTTCCAAAAATGG	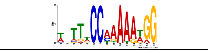	MA0001.1 (AGL3)<br>MA0005.1 (AG)

D

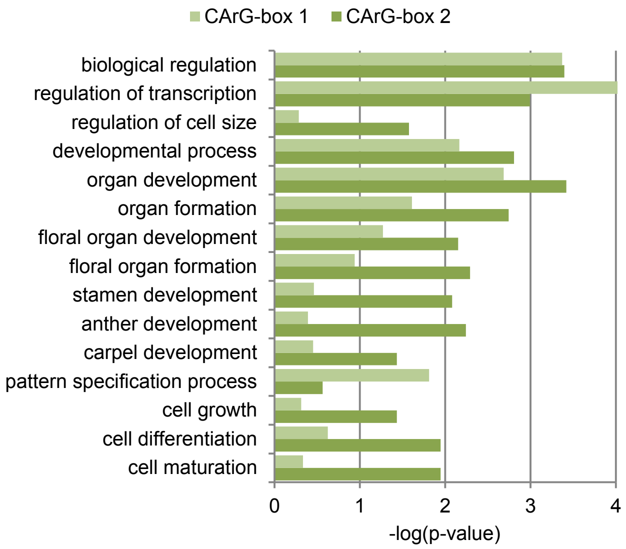

E

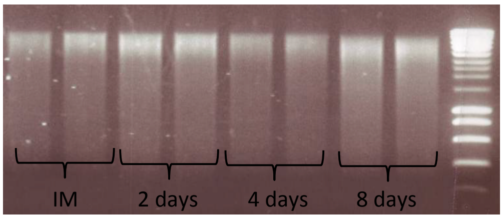

Supplement: Additional file 10: Figure S4 — Chromatin accessibility and TF expression at different stages of flower development. (A) Venn diagram showing the distribution in the expression of cluster 2 and cluster 5 genes with increasing and decreasing DHSs between day 4 and day 8. (B) Venn diagram shows genes with increasing and decreasing DHSs between day 2 vs. 4 and day 4 vs. 8. Forty-six genes have both increasing and decreasing DHSs from day 4 to day 8. (C) Full list of motifs identified by MEME-ChIP in the AP1 and SEP3 peaks regions. Table shows consensus sequences and motifs based on position-specific probability matrices that were identified by MEME-ChIP, and TFs that potentially recognize those motifs identified by TOMTOM. (D) Gene ontology enrichment for SEP3-bound genomic regions at day 8 with CArG-box motif 1 and CArG-box motif 2. The graph shows terminal over-represented categories that belong to ‘biological regulation’ and ‘developmental process’. Only categories with at least five genes and P value <0.05 were considered. € Gel showing partially DNase I-digested chromatin that was submitted for sequencing. [file gb-2014-15-3-r41-S10.pdf]
