# Supplementary material for: Dynamics of chromatin accessibility and gene regulation by MADS-domain transcription factors in flower development
Source: Genome Biol. 2014 Mar 3;15(3):R41. doi: 10.1186/gb-2014-15-3-r41 (PMC4054849; doi:10.1186/gb-2014-15-3-r41)
Supplement: Additional file 12: Figure S5 — Change in MADS-DNA binding precedes change in chromatin accessibility. The figures were obtained in the same way as for Figure 6B. The analysis was repeated for each replicate independently and for the combined analysis for both AP1 (A) and SEP3 (B). The results and conclusions are similar in all cases. [file gb-2014-15-3-r41-S12.pdf]

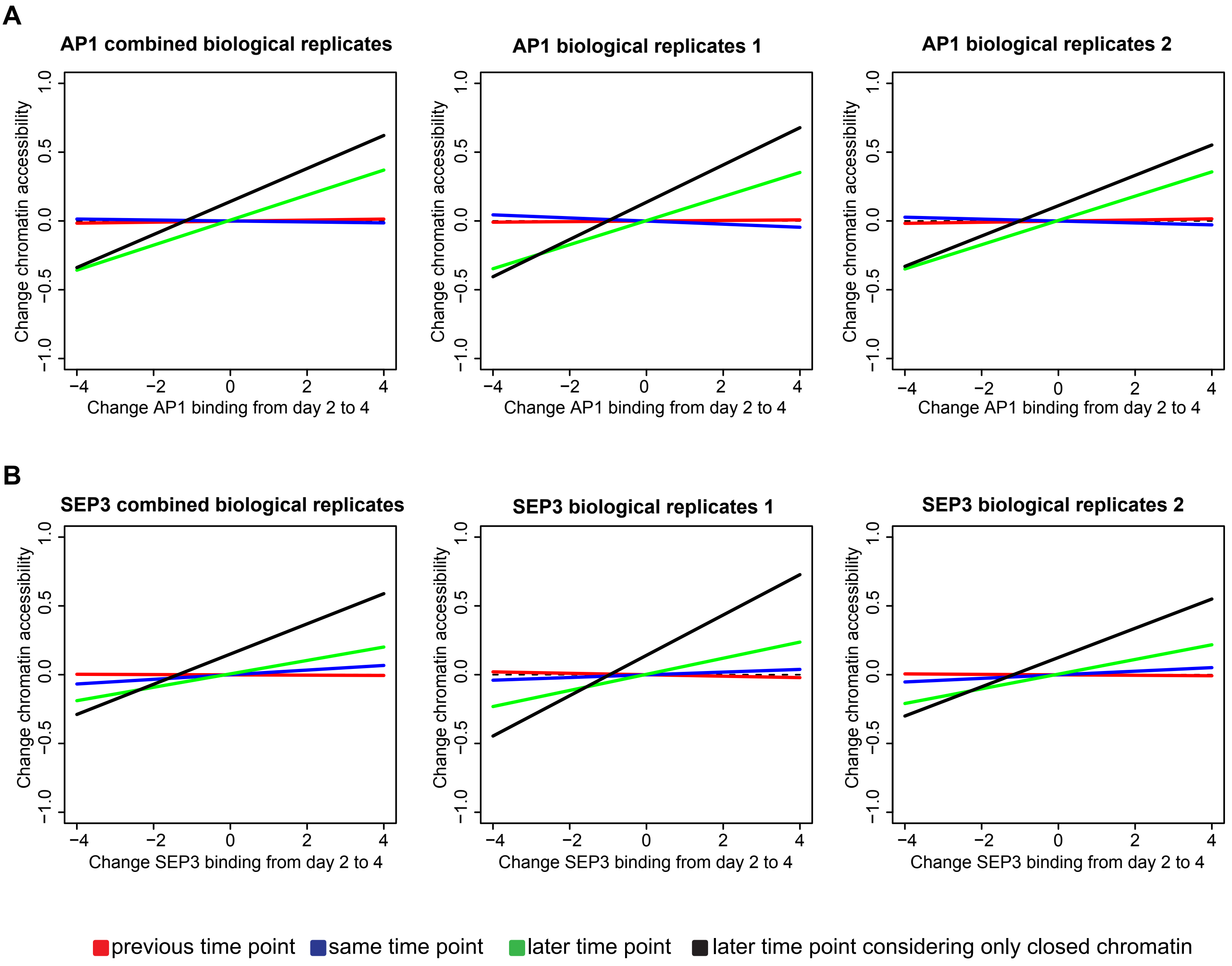
A
AP1 combined biological replicates
AP1 biological replicates 1
AP1 biological replicates 2
Change chromatin accessibility
Change AP1 binding from day 2 to 4
B
SEP3 combined biological replicates
SEP3 biological replicates 1
SEP3 biological replicates 2
Change chromatin accessibility
Change SEP3 binding from day 2 to 4
previous time point
same time point
later time point
later time point considering only closed chromatin
